# Coping strategies and the Salutogenic Model in future oral health professionals

**DOI:** 10.1186/s12909-016-0740-z

**Published:** 2016-08-26

**Authors:** Karla Gambetta-Tessini, Rodrigo Mariño, Mike Morgan, Vivienne Anderson

**Affiliations:** 1 Melbourne Dental School, The University of Melbourne, 5th floor, 720 Swanston St, Parkville, 3010 Melbourne, VIC Australia; 2 Higher Education Development Centre, University of Otago, Dunedin, New Zealand

**Keywords:** Australia, Chile, Coping skills, Dental, New Zealand

## Abstract

**Background:**

Attention to the role of context in shaping individuals’ coping strategies is necessary. This study used the Salutogenic Model (SM) as a framework to identify the coping strategies of oral health profession students from three countries.

**Methods:**

Students from Australia, New Zealand and Chile were invited to participate in this cross-sectional study, and were given a questionnaire including socio-demographics, the Perceived Stress Scale, The SOC-13 and the Brief COPE. Descriptive analysis, correlation analysis and profile analysis were computed using SPSS v 20.0.

**Results:**

Eight-hundred and ninety-seven valid questionnaires were returned, achieving a 44 % response rate. The coping dimension that the participants most commonly reported using was “Active Coping” with a mean value of 5.9 ± 1.5. Chilean respondents reported higher stress levels (19.8 vs. 17.7) and a lower Sense of Coherence (55.6 vs. 58.0) compared to Australian/New Zealand participants (*p* < 0.001). The SOC was positively correlated with active coping (*p* < 0.01) and positive reframing (*p* < 0.01). Profile analysis showed that when the differences in responses by sex were accounted for, there was no significant effect by country on the coping strategies used (*p* < 0.32).

**Conclusion:**

This initial investigation provides insights into the students’ coping strategies and the validity of the SM. Students reporting high SOC scores where those who demonstrated the use of active coping and positive reframing as strategies to deal with stressful situations, which indicates the accuracy of the theoretical framework of the SM in health education environments. The results also suggest that a distinctive coping strategy pattern may apply to all participants, regardless of their country and sex.

**Electronic supplementary material:**

The online version of this article (doi:10.1186/s12909-016-0740-z) contains supplementary material, which is available to authorized users.

## Background

Coping strategies are conscious efforts used by individuals to solve everyday problems, demands and conflicts [1]. These strategies are highly moderated by the environment, in particular when the person has to deal with obstacles and impediments to fulfil their goals [2]. Coping strategies can be classified in many ways. Nonetheless, the Salutogenic Model (SM) is a particularly useful framework for evaluating psycho-social characteristics. This model proposes that challenging situations and conflicts are characteristically inherent to the human condition [3]. The SM focus is on how individuals effectively manage and cope with adverse situations to preserve their health [3]. The salutogenic potential of individuals is represented by the Sense of Coherence (SOC), which is “the capability to perceive that one can manage in any situation independent of whatever is happening in life” [4]. In the SM, coping strategies help in shaping and increasing the person’s salutogenic potential; in other words, they can be protective against the damaging influences on health of psychological demands [5], such as psychological distress, depression, anxiety and burnout [6].

Medical, nursing and dental education environments have been described as stressful for students. Stress-related disorders reported amongst oral health professionals may develop during the early stages of their careers [7]. The literature indicates that university years are critical in developing good coping strategies, which are likely to buffer later work-related challenges and occupational stress [8]. Unfortunately, this issue has received limited research or policy attention. Furthermore, students are rarely introduced to strategies for coping with professional pressures, constant worries and demands, as part of their university programs [9].

There is little research available on SM and coping in health education environments. Research on the sources of stress in Australian contexts indicates that governmental regulatory pressures and examinations are the main stressors for oral health professionals and students, respectively [10, 11]. Nonetheless, these reports have been largely restricted to analyses of singular stress data collected within the boundaries of the nation. The salutogenic role of coping strategies and the influence of culture has not been fully investigated. Attention to the role of the cultural context in shaping coping behaviours may facilitate a better understanding of how to foster coping behaviours across nations, and the value of health education and health promotion programs aimed at health professionals across a range of cultural contexts. As a starting point in the exploration of coping mechanisms within health professional environments, this cross-cultural study was undertaken among oral health profession students in Australian/New Zealand and Chile to identify their coping strategies and their relationships with SOC and perceived stress levels. The present study expands on previous research and examines a critical question: do students’ coping strategies differ depending on their cultural backgrounds or do broad patterns of coping exist amongst them regardless of the cultural context?

Attention to individual coping strategies that improve individuals’ salutogenic potential (i.e., SOC) may inform the development of policies and education programs aimed at promoting good coping techniques amongst students.

## Methods

### Participants and procedures

This study was part of a larger investigation in a multinational sample of oral health profession students [12]. Participants included all students (*n* = 2049) aged 18-years-old and older at two Australian universities - The University of Queensland (UQ) and The University of Sydney (US), one New Zealand university - The University of Otago (UO), and two Chilean universities - The University of Talca (UT) and The University of Valparaiso (UV). The selection of schools was partly due to convenience. However, these countries were also selected for cultural reasons. Australia and New Zealand, despite their diverse ethnic composition [13], like other Western countries, place responsibility on the individual and individual-centred programs, empowerment and personal enrichment [11]. In contrast, countries like Chile are characterised by a more hierarchical societal structure, and a focus on the wellbeing of the group rather than individuals [14].

Ethics approval was obtained from The University of Melbourne HREC (#0932899) and from all participating universities. Data collection was extended to May 2011. During a regularly scheduled class meeting, all students from first to final year were briefed on the aims of the study and invited to participate. Participants were asked to voluntarily complete the anonymous questionnaire in their own time and to return it as soon as possible. There were two-week and four-week follow-up reminders.

### Instruments

The questionnaire included:

1. Socio-demographic information: age, sex and high school attended (private vs. public); and country, categorised as a) Australia/New Zealand and b) Chile.
2. The Perceived Stress Scale (PSS): to establish the degree to which situations in one’s life are appraised as stressful over a one-month period [15]. The PSS is a 10-item instrument with scores ranging from 0 to 40. High scores indicate high stress levels. The internal reliability value for this scale was 0.85 [12].
3. The Orientation to Life Questionnaire (SOC-13): The Sense of Coherence was measured by the SOC-13. Scores of the SOC-13 ranged from low 13 to high 91 [3]. Higher scores indicate stronger SOC, with a Cronbach’s alpha of 0.79 [12].
4. The Brief Coping Orientation for Problems Experienced [16]: The Brief (COPE) scale has been utilised with Australian tertiary students [17], and a valid Spanish version is available [18]. Coping strategies were identified as *Adaptive* and *Maladaptive* [19]. The Brief COPE consists of fourteen dimensions with two items each (28 items in total), including adaptive coping strategies (e.g., active coping, planning, positive reframing, acceptance, humour, religion, seeking emotional and instrumental support) and maladaptive coping techniques (e.g., self-distraction, denial, venting, substance use, behavioural disengagement, and self-blame). The response options ranged from 1 (*I have not been doing this at all*) to 4 (*I have been doing this a lot*). Adaptive coping ranges from a score of 16 to 64, and maladaptive coping ranges from 12 to 48. The internal reliability value of the scale was 0.84 [12].

### Data analysis

The analysis provided descriptive information on the participants’ characteristics, coping items, SOC and stress levels. To identify the association between study variables and socio-demographic characteristics data analysis included bivariate analysis (i.e., ANOVA and chi-square). To identify the relationship between SOC, stress and coping strategies, Pearson’s correlation was used. In the last part of the analysis, a special application of multivariate analysis of variance (MANOVA) called profile analysis was performed to test the main effect of country and sex upon each individual coping dimension. This profile analysis was also used to explore coping strategies’ mean values by the interaction of country with sex. Due to the exploratory nature of this study, the significance criterion was set at 0.05. Data were handled and analysed using SPSS V 20.0.

## Results

Nine survey packages were returned incomplete. A total of eight hundred and ninety-seven participants returned valid questionnaires, achieving a 44 % response rate. Of those, 66.8 % (*n* = 599) were from Chile and 33.2 % (*n* = 298) were from Australian/New Zealand universities.

Participants’ socio-demographic characteristics are described elsewhere [12]. In brief, participants’ ages ranged from 18 to 38 years old with a mean value of 22.1 ± 2.7 years. The majority were females (59.3 %; *n* = 531). While the majority of Chilean participants came from private schools (78.6 %), the majority of Australian/New Zealand students came from public secondary schools (60.4 %). The largest group of participants lived with their parents (41.2 %; *n* = 340) with significant differences by country (*χ*^2^(1) = 6.66; *p* = 0.006). Chileans tended to live with their parents more often than Australian/New Zealand participants (44.2 % vs. 35.2 %).

Participants showed a mean stress level score of 19.1 ± 7.0 for perceived stress level with PSS scores ranging from 0 to 40. Statistically significant differences were found by sex and country. Females reported higher stress levels than men (19.9 vs. 17.8; *p* < 0.001) and Chilean participants reported a higher level of stress than Australian/New Zealand respondents (19.8 vs. 17.6; *p* < 0.001).

Participants reported a mean SOC value of 56.4 ± 10.9 with scores ranging from 17 to 91. Statistically significant differences were found only by country. Chilean participants reported lower SOC than Australian/New Zealand students (55.6 vs. 58.0; *p* < 0.05).

### Coping strategies

Table 1 illustrates coping strategies, SOC and perceived stress mean values by respondents’ socio-demographic characteristics. The coping dimension that the participants reported using most commonly was “Active Coping” with a mean value of 5.9 ± 1.5. The least reported strategy dimension was “Substance Use” with a mean value of 2.8 ± 1.4. Females reported using significantly less substance use (2.6 vs. 3.1; *p* < 0.001) and humour (4.4 vs. 4.7; *p* < 0.05) as coping strategies than males. In contrast, males reported significantly less use of emotional support (5.0 vs. 5.9; *p* < 0.001), instrumental support (4.9 vs. 5.7; *p* < 0.001) and religion (4.0 vs. 4.5; *p* < 0.001) as strategies for coping with stress. Statistically significant differences were found in all coping dimensions between Australian/New Zealand and Chilean participants. Overall, Chilean respondents reported using more adaptive (44.8 vs. 38.1; *p* < 0.001) and maladaptive coping techniques (25.4 vs. 21.0; *p* < 0.001). Religion-associated items, emotional and instrumental support, humour, substance use, behavioural disengagement, and venting showed significant statistical differences by all selected independent variables (i.e., sex, country, high school attended).

**Table 1:** Summary of mean (s.d) for SOC, perceived stress, coping dimensions by socio-demographic characteristics

		SOC	Perceived stress	Adaptive coping dimensions								Maladaptive coping dimensions						Type of coping strategy	
				Active coping	Emotional support	Instrumental support	Positive reframing	Planning	Humour	Acceptance	Religion	Self-distraction	Denial	Substance use	Behavioural disengagement	Venting	Self-blame	Adaptive	Maladaptive
*Total (897)*		56.4 (10.9)	19.1 (7.0)	5.9 (1.5)	5.5 (1.8)	5.4 (1.9)	5.5 (1.6)	5.8 (1.5)	4.5 (1.8)	5.7 (1.4)	4.3 (2.1)	5.6 (1.5)	2.9 (1.4)	2.8 (1.4)	3.2 (1.4)	4.7 (1.7)	4.9 (1.7)	42.7 (8.7)	24.1 (5.5)
*Sex n (%)*	Female 531*(59.3)*	56.1 (11.1)	19.9 (6.9)**	6.0 (1.4)	5.9 (1.7)**	5.7 (1.8)**	5.6 (1.6)*	5.9 (1.6)	4.3 (1.8)*	5.7 (1.4)	4.5 (2.2)**	5.6 (1.5)	3.0 (1.4)	2.6 (1.3)**	3.2 (1.4)	4.8 (1.6)*	5.0 (1.7)	43.7 (8.5)**	24.2 (5.2)
	Male *364 (40.7)*	56.8 (10.7)	17.9 (6.9)**	5.9 (1.4)	5.0 (1.8)**	4.9 (1.9)**	5.3 (1.6)*	5.7 (1.5)	4.7 (1.8)*	5.6 (1.5)	4.0 (2.0)**	5.5 (1.5)	2.9 (1.3)	3.1 (1.6)**	3.2 (1.4)	4.5 (1.7)*	4.9 (1.7)	41.1 (8.8)**	23.9 (5.8)
*Country n (%)*	Australia/New Zealand *298 (33.2)*	58.0 (11.3)*	17.7 (6.8)**	5.5 (1.4)**	4.7 (1.7)**	4.6 (1.7)**	5.1 (1.6)**	5.3 (1.5)**	4.0 (1.8)**	5.5 (1.5)*	3.5 (1.8)**	5.1 (1.6)**	2.5 (1.0)**	2.6 (1.3)*	2.8 (1.1)**	3.9 (1.5)**	4.3 (1.6)**	38. 1 (8.1)**	21.0 (5.0)**
	Chile *599 (66.8)*	55.6 (10.7)*	19.8 (7.0)**	6.2 (1.3)**	5.9 (1.8)**	5.8 (1.8)**	5.7 (1.6)**	6.0 (1.5)**	4.7 (1.8)**	5.8 (1.4)*	4.7 (2.1)**	5.8 (1.4)**	3.2 (1.5)**	2.9 (1.5)*	3.4 (1.4)**	4.9 (1.7)**	5.3 (1.7)**	44.8 (8.1)**	25.4 (5.2)**
*Previous education n (%)*	Public *305 (34.3)*	56.9 (11.0)	18.5 (7.1)	5.8 (1.4)*	5.1 (1.8)**	4.9 (1.9)**	5.3 (1.6)	5.6 (1.6)*	4.3 (1.9)*	5.5 (1.5)*	3.8 (2.0)**	5.4 (1.6)	2.9 (1.3)	2.6 (1.3)*	3.0 (1.2)*	4.4 (1.6)*	4.8 (1.7)*	40.2 (9.5)**	23.1 (5.6)**
	Private *583 (65.7)*	56.0 (10.9)	19.4 (6.9)	6.1 (1.4)*	5.7 (1.8)**	5.6 (1.8)**	*5.5 (1.6)*	5.9 (1.5)*	4.6 (1.8)*	5.8 (1.4)*	4.6 (2.1)**	5.6 (1.4)	3.0 (1.4)	2.9 (1.5)*	3.3 (1.4)*	4.8 (1.7)*	5.1 (1.5)*	43.8 (8.3)**	24.6 (5.5)**

SOC Sense of Coherence, *Significant values *p* < 0.05, ** Significant values *p* <0.001. Results may not add due to missing values

Statistically significant positive correlations were found between stress levels and most maladaptive coping techniques, particularly self-blame (*r* = 0.42; *p* < 0.01). On the other hand, active coping (*r* = -0.1; *p* < 0.01) and positive reframing (*r* = -0.13; *p* < 0.01) showed a significant negative correlation with perceived stress. Interestingly, some adaptive-classified dimensions correlated positively with stress levels. These included religion (*r* = 0.11; *p* < 0.01), emotional (*r* = 0.15; *p* < 0.01), and instrumental support (*r* = 0.15; *p* < 0.01).

The coping strategies that correlated positively with the SOC were active coping (*r* = 0.14; *p* < 0.01) and positive reframing (*r* = 0.13; *p* < 0.01). Instrumental support negatively correlated with the SOC (*r* = -0.08; *p* < 0.05). All maladaptive coping strategies were negative correlated with the SOC (*p* < 0.01). Table 2 demonstrates these findings.

**Table 2:** Correlation matrix between sense of coherence, perceived stress and coping dimensions

	SOC	Adaptive coping dimensions								Maladaptive coping dimensions						Type of coping strategy	
		Active coping	Emotional support	Instrumental support	Positive reframing	Planning	Humour	Acceptance	Religion	Self-distraction	Denial	Substance use	Behavioural disengagement	Venting	Self-blame	Adaptive	Maladaptive
*SOC*	1	0.14 **	−0.06	−0.08*	0.13**	0.07	0.03	0.01	−0.02	−0.12**	−0.24**	−0.22**	−0.26**	−0.24**	−0.38**	0.03	−0.42**
Perceived stress	−0.61**	−0.1**	0.15**	0.15**	−0.13**	0.004	−0.03	−0.03	0.11**	1.32**	0.25**	0.11**	0.28**	0.25**	0.42**	0.04	0.40**

SOC, sense of coherence. *Significant values *p* < 0.05, ** Significant values *p* < 0.01

### Profile analysis

**Fig. 1:**
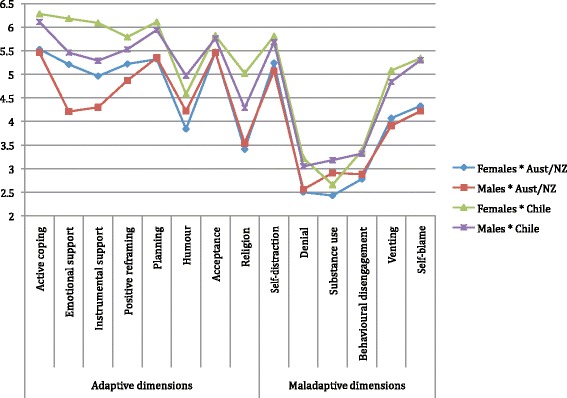
Profile analysis for means of coping dimensions by country and sex interactions

Overall, the mean values of self-reported coping dimensions differed statistically over each of the 14 coping dimensions (*F*(1,853) = 243.44; *p* < 0.001). The statistical model indicated that participants reported higher mean values for adaptive strategies (e.g., active coping and planning) than maladaptive strategies (e.g., denial, substance use, and behavioural disengagement) (See Fig. 1). Of the maladaptive strategies, self-distraction 5.6 ± 1.5, self-blame 4.9 ± 1.7 and venting 4.7 ± 1.7 had the highest mean responses.

Profile analyses showed the independent effect of sex and country on the coping strategies reported by participants. The mean values for coping strategies were significantly different by country (*F*(1, 853) = 6.679; *p* < 0.001). The analysis also indicated an independent effect of sex (*F*(1, 853) = 7.331; *p* < 0.001).

Nevertheless, when the interaction of country with sex was tested, there were no significant differences between groups (i.e., Australian/New Zealand females, Australian/New Zealand males, Chilean females, and Chilean males) indicating that the groups’ overall pattern of responses do not deviate significantly from parallelism (*F*(1,853) = 3.121; *p* = 0.10) (See Fig. 1). The mean value of responses for each coping dimension on Fig. 1 shows that the greatest gap between groups was in the use of emotional and instrumental support. However, despite differences in these two dimensions, this analysis indicated that there were similar mean values in participants’ individual responses regardless of the interaction of country with sex (*F*(1, 853) = 0.980; *p* = 0.32).

## Discussion

The present study showed that students reported the use of adaptive coping strategies as a way of successfully coping with demands, as the most used strategy was active coping. The least reported coping strategy was substance use, a maladaptive strategy, which is consistent with reports reflecting a reduction in smoking and drinking behaviours among dental students [20–22]. However, it has been reported that alcohol consumption remains a concern amongst Australian dentists [23].

The analysis also indicated that participants endorsed different dimensions of coping strategies, relating to adaptive and maladaptive techniques. Independent of country and sex, the profile analysis demonstrated an overall difference in the response of each of the eight adaptive dimensions and six maladaptive dimensions. Specifically, the main difference was found in the participants’ report of using instrumental and emotional support (See Fig. 1). In the SM, social support is counted as an independent psychosocial construct which includes both, a) functioning support; where the person finds instrumental or concrete support provided by others, and b) less concrete, or emotional, support [24]. Social support may improve the ability of the person to obtain meaningful information, resulting in a positive influence on health (i.e., a salutogenic outcome) [3]. Surprisingly, the present results showed that support-related dimensions positively correlated with perceived stress, and in particular, instrumental support negatively correlated with SOC. According to the SM, social support should function along with other resources, such as economic or material resources, intelligence and physiological health, to promote the individual’s perception of the world as more organised and structured. Further investigations of these other resources is warranted in health professional environments [12].

In the present study, the Brief COPE did not ask participants to specify from whom they seek support. However, students’ support sources are likely to include their closest friends or relatives, as previous research has indicated that students receive little support from instructors in dental education contexts [25]. Schools may play a decisive role in developing students’ capacity to cope with conflict and stress [7]. One way of increasing all students’ access to social support would be to ensure the development of school environments where students feel able to seek support from peers, administrative staff and academics as one way of coping with stress [26].

In the bivariate analysis, Australian/New Zealand respondents reported lower mean values for all coping dimensions, either adaptive or maladaptive strategies. However, they also reported a lower stress level and higher SOC compared to Chilean participants. This finding may illustrate the significance of the SOC in moderating individuals’ stress levels [27]. The SOC scores for dental students were lower than those reported in research involving medical students, which could explain why historically, dental students have expressed higher stress levels compared to medical students [28]. In addition no gender differences were reported in the SOC mean values, which is consistent with the Salutogenic theory, indicating that SOC is a construct that is not linked with gender [29]. The major strategies that increased SOC in the present study were active coping and positive reframing. Active coping is the process of making active attempts to remove or avoid conflict or to improve its effects. Positive reframing refers to transforming a demanding circumstance into a positive situation [30]. Both coping strategies ‘fit’ the central component of the SOC - “meaningfulness” - which refers to a person’s capacity to consider demands as positive challenges, and not as problems or troubles [3].

Consistent with other studies involving undergraduate students [31, 32], females tended to report more adaptive coping than males, such as, seeking emotional support, and turning to religion. However, in this study, when we controlled for country and sex, the responses did not differ. Thus, our results suggest that there might exist a single distinctive coping strategy pattern to which all participants subscribe, regardless of their country or sex.

The present findings, however, do not imply that oral health students and the general population within the countries studied show similar coping patterns, or that cultural differences are not important. University students may well be different from the broader population which they are representing, as the coping strategy patterns might be moderated by the kind of principles being promoted by higher education, such as professional skills, knowledge and discipline [33]. Studies have indicated that in courses like dentistry, which involve a large amount of time in class, laboratory, clinical work and library study, students become immersed in the school environment [13]. Therefore, as part of the undergraduates’ professional socialisation, their permanent coping strategies may align with the school/department profile.

As in any study, this study is not without limitations. The most obvious ones are the relatively low response rate, the study’s use of convenience sampling, and its reliance on self-reporting and self-selection. The survey package included the PSS, the Brief COPE and SOC along with socio-demographic questions. The length of the questionnaire may have generated some response fatigue and contributed to the low response rate. In this study, the use of convenience sampling led to an over-representation of Chilean students, which limits the generalisation of the results. Questions such as why and under what conditions participants choose their coping strategies were not addressed by the present study. However, despite the limitations, this initial investigation provides insights into health professional students’ ways of coping with stress, and raises some important questions for future research. For instance, research is needed to ascertain the usefulness of the SM in health professional environments. For example, research could explore whether similar results emerge in countries other than those included in this study and investigate other constructs of the SM. The results of this study provide information which could lead to educational programs tailored to specific individual characteristics, reported coping strategies and apparent areas of need. An international review reported that only four studies demonstrated effective stress reduction in oral health education programs which included attention to relaxation techniques, interpersonal approaches to dentistry, and stress management seminars for dealing with stress [34]. However, research is also necessary to examine prospectively their association with occupational/professional stress. Future research could also explore the relationship between reported stress, SOC and coping strategies in an inter-professional sample, for example, whether students or professionals in allied health programs report similar patterns of coping.

The present cross-sectional study has implications regarding the influence of the cultural context in stress, SOC and coping strategies, which apply, not only to oral health students, but also to university students at large. Longitudinal studies that follow the development of coping skills and other psychosocial constructs from tertiary education to professional life may be of interest.

## Conclusions

Early identification of students’ coping strategies may facilitate the development of interventions that foster adaptive coping strategies to create a more salutogenic environment and reduce stress levels for health professionals during the early stages of their formation. This may facilitate academic and professional success and contribute to the prevention of stress-related consequences, such as depression, anxiety, burnout and psychological distress [34]. The present data would also suggest that efforts to increase adaptive coping strategies could be exchanged across national borders.

## Additional file

Additional file 1: Questionnaire. (DOC 408 kb)

## Abbreviations

COPE: Coping orientation for problems experienced
PSS: Perceived stress scale
SM: Salutogenic model
SOC: Sense of coherence

## References

[1] Lazarus RS, Folkman S (1984). Stress, Appraisal, and Coping.

[2] Carver CS, Connor-Smith J (2010). Personality and Coping. Annu Rev Psychol..

[3] Antonovsky A (1979). Health, Stress, and Coping. New Perspectives on Mental and Physical Well-Being., first edition. edn.

[4] Lindstrom B, Eriksson M (2006). Contextualizing salutogenesis and Antonovsky in public health development. Health Promot Int.

[5] Bernabe E, Kivimaki M, Tsakos G, Suominen-Taipale AL, Nordblad A, Savolainen J, Uutela A, Sheiham A, Watt RG (2009). The relationship among sense of coherence, socio-economic status, and oral health-related behaviours among Finnish dentate adults. Eur J Oral Sci.

[6] Alzahem AM, van der Molen HT, Alaujan AH, Schmidt HG, Zamakhshary MH (2011). Stress amongst dental students: a systematic review. Eur J Dent Educ.

[7] Rada RE, Johnson-Leong C (2004). Stress, burnout, anxiety and depression among dentists. J Am Dent Assoc.

[8] Heiman T (2004). Examination of the salutogenic model, support resources, coping style, and stressors among Israeli university students. J Psychol.

[9] Alexander RE (2001). Stress-related suicide by dentists and other health care workers - Fact or folklore?. J Am Dent Assoc.

[10] Sanders AE, Lushington K (1999). Sources of stress for Australian dental students. J Dent Educ.

[11] Johns RE, Jepsen DM (2015). Sources of occupational stress in NSW and ACT dentists. Aust Dent J..

[12] Gambetta-Tessini K, Mariño R, Morgan M, Evans W, Anderson V (2013). Stress and Health-Promoting Attributes in Australian, New Zealand, and Chilean Dental Students. J Dent Educ.

[13] Mariño RJ (2004). Cultural consistency in Australian dental students from two different ethnic backgrounds. J Dent Educ.

[14] Early P, Erez M (1997). The Transplanted Executive: Why You Need to Understand How Workers in Other Countries See the World Differently.

[15] Cohen S, Kamarck T, Mermelstein R (1983). A global measure of perceived stress. J Health and Soc Behav.

[16] Carver CS (1997). You want to measure coping but your protocol's too long: Consider the brief COPE. Int J Behav Med.

[17] Khawaja NG (2008). A comparison of International and Domestic Tertiary Students in Australia. Aust J Guid Couns.

[18] Perczek R, Carver CS, Price AA, Pozo-Kaderman C (2000). Coping, mood, and aspects of personality in Spanish translation and evidence of convergence with English versions. J Pers Assess.

[19] Grota BL (2005). The relationship among coping strategies, perceived stress, and sense of coherence. Ph.D.

[20] Underwood B, Fox K (2000). A survey of alcohol and drug use among UK based dental undergraduates. Br Dent J.

[21] Underwood B, Fox K, Manogue M (2010). Tobacco, alcohol and drug use among dental undergraduates at one English university in 1998 and 2008. Br Dent J.

[22] Barber MW, Fairclough A (2006). A comparison of alcohol and drug use among dental undergraduates and a group of non-medical, professional undergraduates. Br Dent J.

[23] Winwood PC, Winefield AH, Lushington K (2003). The role of occupational stress in the maladaptive use of alcohol by dentists: A study of South Australian general dental practitioners. Aust Dent J.

[24] Zimet GD, Dahlem NW, Zimet SG, Farley GK (1988). The multidimensional scale of perceived social support. J Pers Assess.

[25] Muirhead V, Locker D (2008). Canadian dental students' perceptions of stress and social support. Eur J Dent Educ.

[26] Lopez N (2010). Does peer mentoring work? Dental students assess its benefits as an adaptive coping strategy. J Dent Educ.

[27] Eriksson M, Lindstrom B (2006). Antonovsky's sense of coherence scale and the relation with health: a systematic review. J Epidemiol Community Health.

[28] Biro E, Balajti I, Adany R, Kosa K (2009). Determinants of mental well-being in medical students. Soc Psychiatry Psychiatr Epidemiol.

[29] Lindmark U, Stenstrom U, Gerdin EW, Hugoson A (2010). The distribution of ''sense of coherence'' among Swedish adults: a quantitative cross-sectional population study. Scand J Public Health.

[30] Carver CS, Scheier MF, Weintraub JK (1989). Assessing coping strategies: a theoretically based approach. J Pers Soc Psychol.

[31] Doron J, Stephan Y, Boiche J, Le Scanff C (2009). Coping with examinations: exploring relationships between students' coping strategies, implicit theories of ability, and perceived control. Br J Educ Psychol.

[32] Dyson R, Renk K (2006). Freshmen adaptation to university life: Depressive symptoms, stress, and coping. J Clin Psychol.

[33] Altbach P, Lewis L (1992). Not to Worry: The College Mold Doesn't Hold. Commonweal Commonweal Foundation..

[34] Alzahem AM, Van der Molen HT, Alaujan AH, De Boer BJ (2014). Stress management in dental students: a systematic review. Adv Med Educ Pract..

