# Supplementary material for: Coping strategies and the Salutogenic Model in future oral health professionals
Source: BMC Med Educ. 2016 Aug 26;16(1):224. doi: 10.1186/s12909-016-0740-z (PMC5000445; doi:10.1186/s12909-016-0740-z)
Supplement: Additional file 1: — Questionnaire. (DOC 408 kb) [file 12909_2016_740_MOESM1_ESM.doc]

| **Please fill-in the number or complete**  **Let's talk about you first**  **any blank spaces to indicate your response**  A.1 How old are you? _______ years old years.  A.2 Sex:  ① Female  ② Male  A.3 Which year of the dental course are you in?  ① First   - Second   ③ Third  ④ Fourth   - Fifth   A.4 What is the name of your University?______________ |  |
| --- | --- |

| **A.5** | | **Last secondary school attended was** |
| --- | --- | --- |
| ① | | Australian State High School |
| ② | | Australian Private High School |
| ③ | | Overseas Public/State High School |
| ④ | Overseas Private High School | |
| ⑤ | | Other, please specify_____________________ |

| **A.6** | | **What will be your main residence while attending University this year?** |
| --- | --- | --- |
| ① | | Parents’ home |
| ② | | Home of other relative or family friend |
| ③ | | Boarding house or lodging |
| ④ | Hostel | |
| ⑤ | | University college |
| ⑥ | | House or flat rented or shared by non family members |
| ⑦ | | Other, Please specify_________________ |

| **A.7** | **As a child, what was the first language that you spoke?** |
| --- | --- |
| ① | English |
| ② | English and ____________ at the same time |
| ③ | Other, ____________ |

| **A.8** | **How well do you understand spoken English?** |
| --- | --- |
| ① | Very well |
| ② | Well |
| ③ | Reasonably well |
| ④ | Not well |

| **A.9** | **How well do you understand written English?** |
| --- | --- |
| ① | Very well |
| ② | Well |
| ③ | Reasonably well |
| ④ | Not well |

| **A.11** | **What is your marital status?** |
| --- | --- |
| ① | Single |
| ② | Married or in a *de-facto* relationship |
| ③ | Separated/divorced |
| ④ | Widowed |
| ⑤ | Other, Please specify_________________ |

| **A.10** | **How do you pay for your studies?** |
| --- | --- |
| ① | University Loan |
| ② | Parents Money |
| ③ | Own Money |
| ④ | Scholarship |
| ⑤ | Other, Please specify________________ |

| **What is your parents’ highest level of formal education?** | | **A.12 Father** | | **A.13 Mother** |
| --- | --- | --- | --- | --- |
| None | | ① | | ① |
| Primary school | | ② | | ② |
| Secondary school or equivalent | | ③ | | ③ |
| Technical/Trade school | ④ | | ④ | |
| Undergraduate tertiary degree (e.g. Bachelor’s degree) | | ⑤ | | ⑤ |
| Postgraduate tertiary degree (e.g. Master, PhD) | | ⑥ | | ⑥ |
| Other, Please specify_________________ | | ⑦ | | ⑦ |

**What is/was your parents’ main occupation?**

**A.14** Father___________________**A.15** Mother___________________

|  |  | Australia | Other, please specify |
| --- | --- | --- | --- |
| **A.16** | Where were you born? | ① | ②_________________ |
| **A.17** | Where was your father born? | ① | ②_________________ |
| **A.18** | Where was your mother born? | ① | ②_________________ |
| **A.19** | Where was your father’s father born? | ① | ②_________________ |
| **A.20** | Where was your father’s mother born? | ① | ②_________________ |
| **A.21** | Where was your mother’s father born? | ① | ②_________________ |
| **A.22** | Where was your mother’s mother born? | ① | ②_________________ |

| **A.23 What is your parents’ postcode?** □ □ □ □. (If you do not remember, please indicate the SUBURB and the STATE________________________________) |
| --- |

| **A.24** | **Country of citizenship** |  | **A.25** | **If NOT an Australian citizen, are you:** |
| --- | --- | --- | --- | --- |
| ① | Australia |  | ① | Australian permanent resident |
| ② | Other, please specify______________ |  | ② | International student |

| **Please fill-in the number or complete**  Let's talk about your choice of  dentistry as a career  **dentistry as a career**  **any blank spaces to indicate your response.**  B.1 Please state your first three preferences for  University studies. Eg. Medicine, law, etc.  ① _________________  ② _________________  ③ _________________  B.2 When did you decide to choose dentistry as a career option?  ① Early in high school  ② Late in high school  ③ After leaving high school  B.3 Did you commence your dental course immediately after leaving high school? school?  ① Yes (please go to question B.4)  ② No If you replied ‘**No’** to B3: |  |
| --- | --- |

B.3.a How many years since you left high school? _______ years.

B.3.b What was your occupation before commencing the dental course? ____________________ (If studying, please state, School, Faculty).

| **Person influencing your selection of dentistry as a career** | | | | | | | |  | |  |
| --- | --- | --- | --- | --- | --- | --- | --- | --- | --- | --- |
|  |  | **Little influence** | | |  | Strong influence | | | | |
| B.4 | Self motivation | | ① | ② | ③ | | ④ | | ⑤ |  |
| B.5 | Father | | ① | ② | ③ | | ④ | | ⑤ |  |
| B.6 | Mother | | ① | ② | ③ | | ④ | | ⑤ |  |
| B.7 | Relative or family friend | | ① | ② | ③ | | ④ | | ⑤ |  |
| B.8 | Family dentist | | ① | ② | ③ | | ④ | | ⑤ |  |
| B.9 | Other person within the dental profession | | ① | ② | ③ | | ④ | | ⑤ |  |
| B.10 | Vocational Counsellor | | ① | ② | ③ | | ④ | | ⑤ |  |
| B.11 | School teacher | | ① | ② | ③ | | ④ | | ⑤ |  |
| B.12 | Other. (Please specify) ______________ | | ① | ② | ③ | | ④ | | ⑤ |  |

| **B.13** | **What is your future career preference** |  | **B.14** | **If you have decided to become specialist which one is your preference?** |
| --- | --- | --- | --- | --- |
| ① | General dentist. |  | ① | Orthodontics |
| ② | Specialist. |  | ② | Oral surgeon |
| ③ | Researcher. |  | ③ | Prosthodontist |
| ④ | Educator |  | ④ | Periodontist |
| ⑤ | Other, please specify_____________ |  | ⑤ | Paediatric Dentistry. |
| ⑥ | Undecided. |  | ⑥ | Oral Medicine |
| **If you have chosen specialist, answer number B.14 otherwise just continue.** | |  | ⑦ | Special Need Dentistry |
|  |  |  | ⑧ | Maxillofacial Radiology |
|  |  |  | ⑨ | Other, please specify________________ |
|  |  |  | ⑩ | Undecided |

| ***Reason for choosing dentistry as a career*** | **Little influence** | |  | Strong influence | |  |
| --- | --- | --- | --- | --- | --- | --- |
| B.15 Desire to work for and with people | ① | ② | ③ | ④ | ⑤ |  |
| B.16 Expected lifestyle perceived within the profession | ① | ② | ③ | ④ | ⑤ |  |
| B.17 Flexible hours | ① | ② | ③ | ④ | ⑤ |  |
| B.18 Desire to work independently | ① | ② | ③ | ④ | ⑤ |  |
| B.19 Monetary advantages of the profession | ① | ② | ③ | ④ | ⑤ |  |
| B.20 Caring for and helping other people | ① | ② | ③ | ④ | ⑤ |  |
| B.21 Desire to have a health care occupation | ① | ② | ③ | ④ | ⑤ |  |
| B.22 Interesting career | ① | ② | ③ | ④ | ⑤ |  |
| B.23 Interest in science | ① | ② | ③ | ④ | ⑤ |  |
| B.24 A career in dentistry will give me enough time to be with my family | ① | ② | ③ | ④ | ⑤ |  |
| B.25 Being your own boss | ① | ② | ③ | ④ | ⑤ |  |
| B.26 Better chance of entering and completing dental course than other courses | ① | ② | ③ | ④ | ⑤ |  |
| B.27 A career in dentistry offers job security | ① | ② | ③ | ④ | ⑤ |  |
| B.28 Dentistry has more regular hours than other | ① | ② | ③ | ④ | ⑤ |  |
| B.29 It is easy for dentists to find employment | ① | ② | ③ | ④ | ⑤ |  |
| B.30 There is not much “on call” work | ① | ② | ③ | ④ | ⑤ |  |
| B.31 Other, please specify_____________ |  ①  | ② | ③ |  ④  | ⑤ |  |

| **B.32** | **In first 2 years after graduation, where would you like to work in? Please choose one.** |
| --- | --- |
| ① | A big city |
| ② | Small city |
| ③ | Country |
| ④ | Other, please specify_______________________ |
| ⑤ | Undecided |

| **B.33** | | **After graduation, would you like to work in;** |
| --- | --- | --- |
| ① | | Private practice. |
| ② | | Public sector |
| ③ | | University |
| ④ | | Private practice and public sector |
| ⑤ | | Private practice and University |
| ⑥ | | Public sector and University |
| ⑦ | | Study further |
| ⑧ | | Other, please specify_______________________________ |
| ⑨ | Undecided | |

**…and a few questions about yourself**

The questions in this scale ask you **Fill-in the number** about your feelings and thoughts during the **LAST MONTH**. In each case, indicate how often you felt or thought a certain way.

You do not have to answer every question if you feel uncomfortable about this.

|  | **Never** | **Almost Never** | **Sometimes** | **Fairly often** | **Very Often** |
| --- | --- | --- | --- | --- | --- |
| C.1 In the last month, how often have you been upset because of something that happened unexpectedly? | **⓪** | **①** | **②** | **③** | **④** |
| C.2 In the last month, how often have you felt that you were unable to control the important things in your life? | **⓪** | **①** | **②** | **③** | **④** |
| C.3 In the last month, how often have you felt nervous and “stressed”? | **⓪** | **①** | **②** | **③** | **④** |
| C.4 In the last month, how often have you felt confident about your ability to handle your personal problems? | **⓪** | **①** | **②** | **③** | **④** |
| C.5 In the last month, how often have you felt that things were going your way? | **⓪** | **①** | **②** | **③** | **④** |
| C.6 In the last month, how often have you found that you could not cope with all the things that you had to do? | **⓪** | **①** | **②** | **③** | **④** |
| C.7 In the last month, how often have you been able to control irritations in your life? | **⓪** | **①** | **②** | **③** | **④** |
| C.8 In the last month, how often have you felt that you were on top of things? | **⓪** | **①** | **②** | **③** | **④** |
| C.9 In the last month, how often have you been angered because of things that were outside of your control? | **⓪** | **①** | **②** | **③** | **④** |
| C.10 In the last month, how often have you felt difficulties were piling up so high that you could not overcome them? | **⓪** | **①** | **②** | **③** | **④** |

There are many ways to try to deal with problems.  These items ask what you have been doing to cope with any problem that you are currently facing.  We are interested in how you have tried to deal with it. Each item says something about a particular way of coping.

We want to know to what extent you have been doing what the item says.  How much or how frequently. Do not answer on the basis of whether it seems to be working or not—just whether or not you are doing it. **Try to rate each item separately in your mind from the others.**

| **Fill-in the number of your answers as true FOR YOU as you can.**  **You do not have to answer every question if you feel uncomfortable about this.** | **I have not been doing this at all.** | **I have been doing this a little bit.** | **I have been doing this**  **a medium amount.** | **I have been doing this a lot.** |
| --- | --- | --- | --- | --- |
| D.1 I’ve been turning to work or other activities to take my mind off things. | **①** | **②** | **③** | **④** |
| D.2 I’ve been concentrating my efforts on doing something about the situation I’m in. | **①** | **②** | **③** | **④** |
| D.3 I’ve been saying to myself “this isn’t real”. | **①** | **②** | **③** | **④** |
| D.4 I’ve been using alcohol or other drugs to make myself feel better. | **①** | **②** | **③** | **④** |
| D.5 I’ve been getting emotional support from others. | **①** | **②** | **③** | **④** |
| D.6 I’ve been giving up trying to deal with it | **①** | **②** | **③** | **④** |
| D.7 I’ve been taking action to try to make the situation better. | **①** | **②** | **③** | **④** |
| D.8 I’ve been refusing to believe that it has happened | **①** | **②** | **③** | **④** |
| D.9 I’ve been saying things to let my unpleasant feelings escape. | **①** | **②** | **③** | **④** |
| D.10 I’ve been getting help and advice from other people. | **①** | **②** | **③** | **④** |
| D.11 I've been using alcohol or other drugs to help me get through it. | **①** | **②** | **③** | **④** |
| D.12 I've been trying to see it in a different light, to make it seem more positive. | **①** | **②** | **③** | **④** |
| D.13 I’ve been criticizing myself. | **①** | **②** | **③** | **④** |
| D.14 I've been trying to come up with a strategy about what to do. | **①** | **②** | **③** | **④** |
| D.15 I've been getting comfort and understanding from someone. | **①** | **②** | **③** | **④** |
| D.16 I've been giving up the attempt to cope. | **①** | **②** | **③** | **④** |
| D.17 I've been looking for something good in what is happening. | **①** | **②** | **③** | **④** |
| D.18 I've been making jokes about it. | **①** | **②** | **③** | **④** |
| D.19 I've been doing something to think about it less, such as going to movies, watching TV, reading, daydreaming, sleeping, or shopping. | **①** | **②** | **③** | **④** |
| D.20 I've been accepting the reality of the fact that it has happened. | **①** | **②** | **③** | **④** |
| D.21 I've been expressing my negative feelings. | **①** | **②** | **③** | **④** |
| D.22 I've been trying to find comfort in my religion or spiritual beliefs. | **①** | **②** | **③** | **④** |
| D.23 I’ve been trying to get advice or help from other people about what to do | **①** | **②** | **③** | **④** |
| D.24 I've been learning to live with it. | **①** | **②** | **③** | **④** |
| D.25 I've been thinking hard about what steps to take. | **①** | **②** | **③** | **④** |
| D.26 I’ve been blaming myself for things that happened. | **①** | **②** | **③** | **④** |
| D.27 I've been praying or meditating. | **①** | **②** | **③** | **④** |
| D.28 I've been making fun of the situation. | **①** | **②** | **③** | **④** |

**How do you feel?...**

Here is a series of questions related to various aspects of our lives. Each question has seven possible answers. **Please FILL IN the number** which best expresses your answer, with numbers 1 and 7 being the extreme answers.

**Please give only one answer to each question.**

**You do not have to answer every question if you feel uncomfortable about this.**

| E.1 Do you have the feeling that you don’t really care about what goes on around you? | | | | | | | | |
| --- | --- | --- | --- | --- | --- | --- | --- | --- |
| **Very seldom or never** | **①** | **②** | **③** | **④** | **⑤** | **⑥** | **⑦** | **Very often** |
|  | | | | | | | | |
| E.2 Has it happened in the past that you were surprised by the behaviour of people whom you thought you knew well? | | | | | | | | |
| **Never happened** | **①** | **②** | **③** | **④** | **⑤** | **⑥** | **⑦** | **Always happened** |
|  | | | | | | | | |
| E.3 Has it happened that people whom you counted on disappointed you? | | | | | | | | |
| **Never happened** | **①** | **②** | **③** | **④** | **⑤** | **⑥** | **⑦** | **Always happened** |
|  | | | | | | | | |
| E.4 Until now your life has had: | | | | | | | | |
| **No clear goals or purposes** | **①** | **②** | **③** | **④** | **⑤** | **⑥** | **⑦** | **Very clear goals and purposes** |
|  | | | | | | | | |
| E.5 Do you have the feeling that you are being treated unfairly? | | | | | | | | |
| **Very Often** | **①** | **②** | **③** | **④** | **⑤** | **⑥** | **⑦** | **Very seldom or never** |
|  | | | | | | | | |
| E.6 Do you have the feeling that you are in an unfamiliar situation and don't know what to do? | | | | | | | | |
| **Very Often** | **①** | **②** | **③** | **④** | **⑤** | **⑥** | **⑦** | **Very seldom or never** |
|  | | | | | | | | |
| E.7 Doing the thing you do every day is: | | | | | | | | |
| **Source of deep pleasure and satisfaction** | **①** | **②** | **③** | **④** | **⑤** | **⑥** | **⑦** | **Source of pain and boredom** |
|  | | | | | | | | |
| E.8 Do you have very mixed-up feelings and ideas? | | | | | | | | |
| **Very Often** | **①** | **②** | **③** | **④** | **⑤** | **⑥** | **⑦** | **Very seldom or never** |
|  | | | | | | | | |
| E.9 Does it happen that you have feelings inside you would rather not feel? | | | | | | | | |
| **Very Often** | **①** | **②** | **③** | **④** | **⑤** | **⑥** | **⑦** | **Very seldom or never** |
|  | | | | | | | | |
| E.10 Many people-even those with a strong character- sometimes feel like sad sacks (losers) in certain situations. How often have you felt this way in the past? | | | | | | | | |
| **Never** | **①** | **②** | **③** | **④** | **⑤** | **⑥** | **⑦** | **Very often** |
|  | | | | | | | | |
| E.11 When something happened, have you generally found that: | | | | | | | | |
| **You overestimated or underestimated its importance** | **①** | **②** | **③** | **④** | **⑤** | **⑥** | **⑦** | **You saw things in the right proportion** |
|  | | | | | | | | |
| E.12 How often do you have the feeling that there's a little meaning in the things you do in your daily life? | | | | | | | | |
| **Very often** | **①** | **②** | **③** | **④** | **⑤** | **⑥** | **⑦** | **Very seldom or never** |
|  | | | | | | | | |
| E.13 How often do you have feelings that you are not sure you can keep under control? | | | | | | | | |
| **Very often** | **①** | **②** | **③** | **④** | **⑤** | **⑥** | **⑦** | **Very seldom or never** |

**Finally,…**

**Are there any other issues that you would like to comment on?**

**__________________________________________________________________________________________________________________________________________________________________________________________________________________**

**Thank you very much for your time and effort in completing this questionnaire.**
